# The Microbiota and Health Promoting Characteristics of the Fermented Beverage Kefir

**DOI:** 10.3389/fmicb.2016.00647

**Published:** 2016-05-04

**Authors:** Benjamin C. T. Bourrie, Benjamin P. Willing, Paul D. Cotter

**Affiliations:** ^1^Agricultural, Food and Nutritional Sciences, University of AlbertaEdmonton, AB, Canada; ^2^Teagasc Food Research CentreFermoy, Ireland; ^3^APC Microbiome InstituteCork, Ireland

**Keywords:** gut microbiota, fermented foods, immunomodulation, metabolic diseases, kefir

## Abstract

Kefir is a complex fermented dairy product created through the symbiotic fermentation of milk by lactic acid bacteria and yeasts contained within an exopolysaccharide and protein complex called a kefir grain. As with other fermented dairy products, kefir has been associated with a range of health benefits such as cholesterol metabolism and angiotensin-converting enzyme (ACE) inhibition, antimicrobial activity, tumor suppression, increased speed of wound healing, and modulation of the immune system including the alleviation of allergy and asthma. These reports have led to increased interest in kefir as a focus of research and as a potential probiotic-containing product. Here, we review those studies with a particular emphasis on the microbial composition and the health benefits of the product, as well as discussing the further development of kefir as an important probiotic product.

## Introduction

Fermented dairy products have long been associated with the ability to confer health benefits in those who regularly consume them, with Ellie Metchnikoff first theorizing that their impact on the bacterial microbiota in the gut contributed to health and long life (Metchnikoff, 1908). Indeed many reportedly probiotic-containing foods come in the form of fermented milk products, such as yogurt, koumis, and kefir, many of which have been consumed for 100s of years (Tamime, 2002; Parvez et al., 2006). Probiotics are live microorganisms which, when administered in adequate amounts, confer a health benefit on the host (Hill et al., 2014). As is the case with the fermented dairy products referred to above, probiotics are consumed in foods containing these organisms in sufficiently large quantities to pass safely to the gastrointestinal tract but can also come in the form of supplements consisting of live organisms such as pills.

Although not as widely popular as other fermented dairy products, such as yogurt and cheese, kefir has been consumed and associated with health benefits for 100s of years; originally by communities in the Caucasian mountains. The beverage itself typically has a slightly viscous texture with tart and acidic flavor, low levels of alcohol, and in some cases slight carbonation. Kefir is traditionally made with cow’s milk but it can be made with milk from other sources such as goat, sheep, buffalo, or soy milk (Ismail et al., 1983; Motaghi et al., 1997; Wszolek et al., 2001; Liu et al., 2006a). One of the features that distinguish kefir from many other fermented dairy products is the requirement for the presence of a kefir grain in fermentation and the presence and importance of a large population of yeasts (Tamime, 2002; Tamang et al., 2016). The aforementioned kefir grains are microbially derived protein and polysaccharide matrices that contain a community of bacterial and fungal species that are essential to kefir fermentation (Garrote et al., 2001; Marsh et al., 2013). Traditionally, fermentation was initiated through the addition of kefir grains, which originally formed during the fermentation of milk, to unfermented milk in a sheep or goat skin bag (Motaghi et al., 1997). Commercial, industrial-scale production rarely utilizes kefir grains for fermentation, but rather uses starter cultures of microbes that have been isolated from kefir or kefir grains in order to provide more consistent products (Assadi et al., 2000). While this industrially produced kefir may have health benefits of its own, research examining such benefits has either not been performed or is not published. Thus, any kefir referred to in this review has been produced in a traditional manner using kefir grains or grain fermented milk as the inoculum. In addition to the microbial population present in kefir, these beverages typically also contain an abundance of fermentation products such as organic acids and multiple volatile flavor compounds including ethanol, acetaldehyde, and diacetyl (Güzel-Seydim et al., 2000). As part of the fermentation process, an exopolysaccharide unique to kefir, kefiran, is produced. Kefiran makes up a large proportion of the kefir grain itself and is also found dissolved in the liquid phase, where it contributes to the rheology and texture of the finished product (La Rivière et al., 1967; Frengova et al., 2002; Rimada and Abraham, 2006).

**FIGURE 1:**
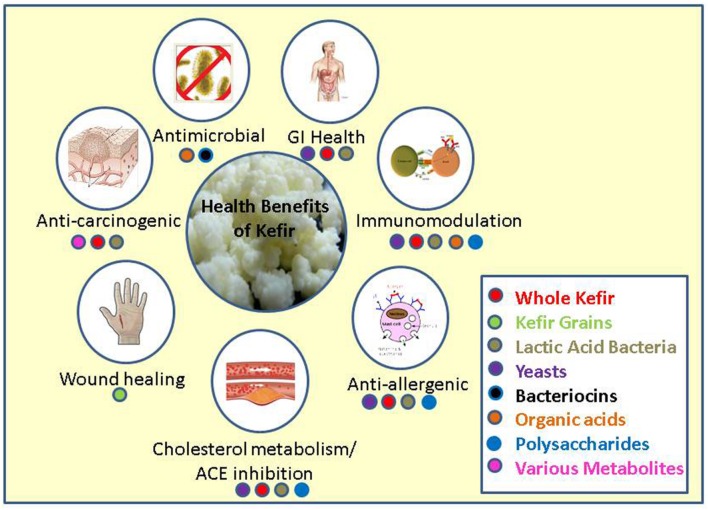
**Major health benefits associated with kefir and the fractions or parts of kefir responsible for these benefits**.

In this review we will discuss the many health promoting effects that have been attributed to kefir, including tumor suppression and prevention, gastrointestinal immunity and allergy, wound healing, cholesterol assimilation and ACE inhibition, its antimicrobial properties, and the ability of kefir to modify the composition and activity of the gut microbiota (**Figure 1**).

## Bacterial and Fungal Populations of Kefir

### Bacterial Populations

**FIGURE 2:**
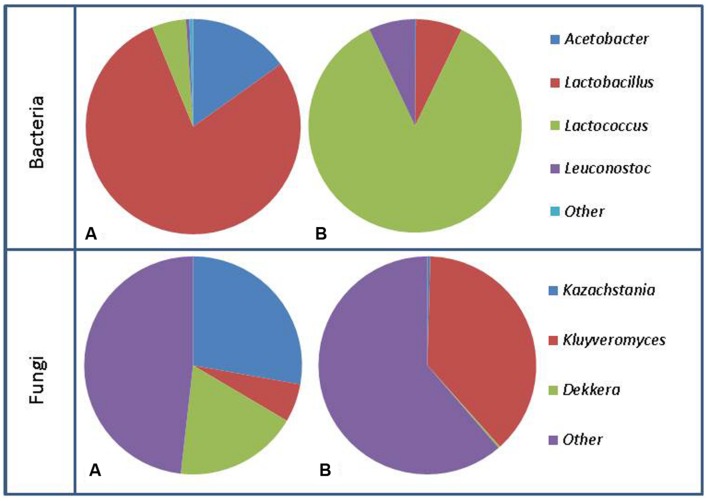
**Representation of bacterial population changes from kefir grain **(A)** to fermented milk **(B)** and fungal population changes from kefir grain **(A)** to fermented milk (B)**. Figure generated using data from Marsh et al. (2013).

Since the first established use, 100s of years ago, the propagation of kefir has been performed by transferring kefir grains from one batch to fresh milk and incubating at ambient temperature. Over this period there has been substantial opportunity for the microbial component of kefir grains to evolve and diverge, resulting in the addition or loss of bacteria and yeasts as well as the addition and loss of genes. The bacterial genera most commonly found in kefir using culture dependent techniques are *Lactobacillus*, *Lactococcus*, *Streptococcus*, and *Leuconostoc* (Simova et al., 2002; Witthuhn et al., 2004; Chen et al., 2008). These genera tend to dominate the population present in both the kefir grain and milk, with *Lactococcus lactis* subsp. *lactis*, *Streptococcus thermophilus*, *Lactobacillus delbrueckii* subsp. *bulgaricus*, *Lactobacillus helveticus*, *Lactobacillus casei* subsp. *pseudoplantarum*, *Lactobacillus kefiri*, *Lactobacillus kefir*, and *Lactobacillus brevis* accounting for between 37 and 90% of the total microbial community present (Simova et al., 2002; Witthuhn et al., 2004; Miguel et al., 2010). While these species commonly make up the majority of the microbial population present in kefir grains, some grains are dominated by yeast species or other bacterial species such as *Leuconostoc mesenteroides* (Witthuhn et al., 2004). The proportions of species can also differ between the grain and milk (**Figure 2**). For example, *L. lactis* subsp. *lactis*, and *S. thermophilus* levels are generally much greater in the fermented kefir than in the kefir grains. The levels of these species increase further in kefir made from kefir as an inoculum. Indeed, the total increase observed has been as much as 30% in some cases (Simova et al., 2002). The reason for this increase during fermentation in the milk may be due to an increase in temperature created by the active fermentation or simply due to where these bacteria reside in the kefir grain, as organisms such as *Lactobacillus* may tend to reside deeper within the kefir grain, thus making it harder for them to escape in to the milk.

In agreement with the majority of culture base studies, investigation of the microbial composition of diverse kefir grains using culture independent techniques found that the overall bacterial populations were for the most part dominated by Firmicutes and Proteobacteria, and kefir milk contained a much higher level of representatives of the *Streptococcaceae* than any other family, (Dobson et al., 2011; Marsh et al., 2013). Based on high-throughput sequencing of 16S genes present in kefir grains and milk, it was established that kefir grains typically have 1 (*Lactobacillus*) or 2 (*Lactobacillus* and *Acetobacter*) dominant bacterial genera (Marsh et al., 2013; Nalbantoglu et al., 2014; Garofalo et al., 2015; Korsak et al., 2015). The most common species of *Lactobacillus* have been *L. kefiranofaciens*, *L. kefiri*, and *L. parakefiri* (Dobson et al., 2011; Leite et al., 2012; Hamet et al., 2013; Vardjan et al., 2013; Nalbantoglu et al., 2014; Garofalo et al., 2015; Korsak et al., 2015). There are many other genera present in these grains; however, they typically represent less than 10% of the community (Leite et al., 2012; Marsh et al., 2013; Nalbantoglu et al., 2014; Garofalo et al., 2015). When milk fermented by these same grains was examined, the relative abundance of the genera present vary much more than in the grain, with *Leuconostoc*, *Lactococcus*, *Lactobacillus*, and *Acetobacter* being the most abundant (Marsh et al., 2013; Korsak et al., 2015). As has previously been stated, bacteria found at lower abundance in the kefir grain can become dominant, as species such as *Lactococcus* are minimally represented in kefir grain, but regularly become the most abundant genus present in the kefir milk (Dobson et al., 2011; Marsh et al., 2013). This observation is consistent with past culture based work, where *Lactococcus* was found to increase through the fermentation process (Simova et al., 2002). At the species level, high throughput 16S analysis showed the number of OTUs vary from 24 to 56 in the kefir grain, and 22 to 61 in kefir milk, i.e., much higher than what has been observed utilizing culture dependent techniques (Marsh et al., 2013). These findings highlight the need for future studies to examine the kefir grain and fermented milk rather than the previous tendency to focus solely on the population of the grain.

**FIGURE 3:**
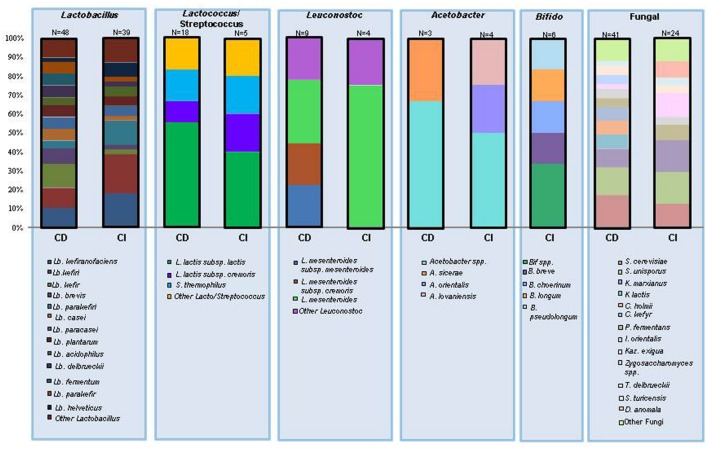
**The number of times an individual species has been identified in kefir expressed as a percentage of the total number of species in the same genera.** CD, culture dependent identification; CI, culture independent identification; *N* values represent the total number of times a species within the genus has been identified.

With respect to the non-lactic acid bacteria (LAB) that have been associated with kefir, it is notable that culture independent methods have revealed *Acetobacter* as one of the dominant genera present in grains. This is of interest as *Acetobacter* is not commonly isolated from kefir *via* culture dependent techniques and, indeed, has been described as a non-essential contaminant of kefir (Angulo et al., 1993; Pintado et al., 1996; Rea et al., 1996; Witthuhn et al., 2004). While there are some studies that have found acetic acid bacteria in large quantities in kefir grains (Rea et al., 1996), many rely on isolation media that is not optimal for growth of acetic acid bacteria without further tests in order to gather an accurate identification (Witthuhn et al., 2005). *Bifidobacterium* species have also been identified through culture independent studies, however, *Bifidobacterium* has not been found in any culture based studies of the kefir microbiota (Dobson et al., 2011; Taş et al., 2012; Marsh et al., 2013). **Table 1** contains a complete list of bacterial species found in both culture dependent and culture independent studies, while **Figure 3** provides a breakdown of the distribution of species found in these studies.

**Table 1:** List of bacterial and fungal species found in kefir grains and milk using both culture dependent and culture independent techniques.

Microbial species	Reference
***Lactobacillus***	
*Lactobacillus kefir*	Angulo et al., 1993; Pintado et al., 1996; Garrote et al., 2001; Santos et al., 2003; Mainville et al., 2006; Miguel et al., 2010
*Lactobacillus kefiranofaciens*	Santos et al., 2003; Mainville et al., 2006; Chen et al., 2008; Dobson et al., 2011; Hamet et al., 2013; Vardjan et al., 2013; Nalbantoglu et al., 2014; Garofalo et al., 2015; Korsak et al., 2015; Zanirati et al., 2015
*Lactobacillus delbrueckii*	Simova et al., 2002; Santos et al., 2003; Witthuhn et al., 2004; Nalbantoglu et al., 2014
*Lactobacillus helveticus*	Simova et al., 2002; Chen et al., 2008; Dobson et al., 2011; Nalbantoglu et al., 2014
*Lactobacillus casei*	Angulo et al., 1993; Simova et al., 2002; Nalbantoglu et al., 2014; Zanirati et al., 2015
*Lactobacillus kefiri*	Chen et al., 2008; Miguel et al., 2010; Dobson et al., 2011; Hamet et al., 2013; Vardjan et al., 2013; Nalbantoglu et al., 2014; Garofalo et al., 2015; Korsak et al., 2015; Zanirati et al., 2015
*Lactobacillus brevis*	Angulo et al., 1993; Simova et al., 2002; Santos et al., 2003; Witthuhn et al., 2005; Nalbantoglu et al., 2014
*Lactobacillus paracasei*	Santos et al., 2003; Miguel et al., 2010; Hamet et al., 2013; Nalbantoglu et al., 2014
*Lactobacillus parakefir*	Takizawa et al., 1994; Garrote et al., 2001; Miguel et al., 2010
*Lactobacillus plantarum*	Garrote et al., 2001; Santos et al., 2003; Miguel et al., 2010; Nalbantoglu et al., 2014
*Lactobacillus satsumensis*	Miguel et al., 2010; Zanirati et al., 2015
*Lactobacillus curvatis*	Witthuhn et al., 2004
*Lactobacillus fermentum*	Angulo et al., 1993; Witthuhn et al., 2004, 2005
*Lactobacillus viridescens*	Angulo et al., 1993
*Lactobacillus acidophilus*	Angulo et al., 1993; Santos et al., 2003; Dobson et al., 2011; Nalbantoglu et al., 2014
*Lactobacillus gasseri*	Angulo et al., 1993; Nalbantoglu et al., 2014
*Lactobacillus kefirgranum*	Takizawa et al., 1994; Vardjan et al., 2013
*Lactobacillus parakefiri*	Dobson et al., 2011; Hamet et al., 2013; Vardjan et al., 2013; Nalbantoglu et al., 2014; Korsak et al., 2015
*Lactobacillus parabuchneri*	Dobson et al., 2011; Nalbantoglu et al., 2014
*Lactobacillus garvieae*	Dobson et al., 2011
*Lactobacillus buchneri*	Nalbantoglu et al., 2014; Garofalo et al., 2015
*Lactobacillus sunkii*	Nalbantoglu et al., 2014; Garofalo et al., 2015
*Lactobacillus crispatus*	Nalbantoglu et al., 2014; Garofalo et al., 2015
*Lactobacillus otakiensis*	Nalbantoglu et al., 2014; Garofalo et al., 2015
*Lactobacillus instestinalis*	Garofalo et al., 2015
*Lactobacillus amylovorus*, *L. pentosus*, *L. salivarius*, *L. johnsonii*, *L. rhamnosus*, *L. rossiae*, *L. sakei*, *L. reuteri*, *L. kalixensis*, *L. rapi*, *L. diolivorans*, *L. parafarraginis*, *L. gallinarum*, *Pediococcus claussenii*, *P. damnosus*, *P. halophilus*, *P. pentosaceus*, *P. lolii*	Nalbantoglu et al., 2014
***Lactococcus/Streptococcus***	
*Lactococcus lactis* subsp. *lactis*	Angulo et al., 1993; Pintado et al., 1996; Garrote et al., 2001; Simova et al., 2002; Witthuhn et al., 2004, 2005; Yüksekdağ et al., 2004; Mainville et al., 2006; Chen et al., 2008; Garofalo et al., 2015; Zanirati et al., 2015
*Lactococcus lactis* subsp. *cremoris*	Yüksekdağ et al., 2004; Mainville et al., 2006; Korsak et al., 2015
*Lactococcus lactis* subsp. *lactis* biovar *diacetylactis*	Garrote et al., 2001
*Lactococcus garvieae*	Nalbantoglu et al., 2014
*Streptococcus salivarius* subsp. *thermophilus*	Angulo et al., 1993
*Streptococcous thermophilus*	Simova et al., 2002; Yüksekdağ et al., 2004; Mainville et al., 2006; Garofalo et al., 2015
*Streptococcus durans*	Yüksekdağ et al., 2004
***Leuconostoc/Oenococcus***	
*Leuconostoc* spp.	Angulo et al., 1993
*Leuconostoc mesenteroides* subsp. *mesenteroides*	Witthuhn et al., 2004; Mainville et al., 2006
*Leuconostoc mesenteroides* subsp. *cremoris*	Witthuhn et al., 2005; Mainville et al., 2006
*Leuconostoc mesenteroides*	Simova et al., 2002; Chen et al., 2008; Nalbantoglu et al., 2014; Korsak et al., 2015; Zanirati et al., 2015
*Leuconostoc pseudomesenteroides*	Mainville et al., 2006
*Oenococcus oeni*	Nalbantoglu et al., 2014
***Acetobacter***	
*Acetobacter* spp.	Angulo et al., 1993; Garrote et al., 2001; Marsh et al., 2013; Garofalo et al., 2015
*Acetobacter sicerae*	Li et al., 2014
*Acetobacter orientalis*, *Acetobacter lovaniensis*	Korsak et al., 2015
***Bifidobacterium***	
*Bifidobacterium* spp.	Marsh et al., 2013
*Bifidobacterium breve*, *B. choerinum, B. longum*, *B. pseudolongum*	Dobson et al., 2011
**Yeast and fungal species**	
*Zygosaccharomyces* spp.	Witthuhn et al., 2004, 2005
*Candida kefyr*	Angulo et al., 1993; Marquina et al., 2002; Witthuhn et al., 2004
*Candida lipolytica*	Witthuhn et al., 2004
*Saccharomyces cerevisiae*	Angulo et al., 1993; Marquina et al., 2002; Simova et al., 2002; Witthuhn et al., 2004; Latorre-García et al., 2007; Marsh et al., 2013; Vardjan et al., 2013; Diosma et al., 2014; Garofalo et al., 2015
*Candida holmii*	Angulo et al., 1993; Witthuhn et al., 2004; Latorre-García et al., 2007
*Torulaspora delbrueckii*	Angulo et al., 1993; Vardjan et al., 2013
*Saccharomyces unisporus*	Angulo et al., 1993; Pintado et al., 1996; Marquina et al., 2002; Latorre-García et al., 2007; Wang et al., 2008; Marsh et al., 2013; Vardjan et al., 2013; Diosma et al., 2014; Garofalo et al., 2015
*Candida friedrichii*	Angulo et al., 1993
*Kluyveromyces lactis*	Angulo et al., 1993; Marquina et al., 2002; Latorre-García et al., 2007
*Pichia fermentans*	Angulo et al., 1993; Wang et al., 2008; Marsh et al., 2013
*Issatchenkia orientalis*	Latorre-García et al., 2007; Marsh et al., 2013; Diosma et al., 2014
*Kluyveromyces marxianus*	Marquina et al., 2002; Wang et al., 2008; Marsh et al., 2013; Vardjan et al., 2013; Diosma et al., 2014; Korsak et al., 2015
*Saccharomyces turicensis*	Wang et al., 2008; Garofalo et al., 2015
*Dekkera anomala*	Marsh et al., 2013; Garofalo et al., 2015
*Kazachstania exigua*	Vardjan et al., 2013; Garofalo et al., 2015; Korsak et al., 2015
*Naumovozyma* spp.	Korsak et al., 2015
*Cryptococcus humicolus*, *Geotricum candidum*	Witthuhn et al., 2005
*Kazachstania servazzii*, *Ka. solicola*, *Ka. aerobia*, *Saccharomyces cariocanus*	Garofalo et al., 2015
*Kluyveromyces marxianus* var. *lactis*, *Candida inconspicua, C. maris*	Simova et al., 2002
*Saccharomyces humaticus*, *Candida sake, Yarrowia lipolytica, Dipodascus capitatus, Trichosporon coremiiforme*	Latorre-García et al., 2007
*Ganoderma lucidum*, *Dioszegia hungarica*, *Heterbasidion annosum*, *Peziza campestris*, *Cyberlindnera jadinii*, *Malassezia pachydermatis*, *Teratosphaeria knoxdaviesii*, *Cryptococcus* sp. *Vega* 039, *Microdochium nivale*, *Wallemia sebi*, *Zygosaccharomyces lentus*, *Eurotium amsteldami*, *Dekkera bruxellensis*, *Kazachstania barnettii*, *Naumovozyma castelli*, *Davidiella tassiana*, *Penicillium* sp. *Vega* 347	Marsh et al., 2013

### Yeast Populations

In addition to the large and variable bacterial population in kefir grains, there is an abundant yeast population that exists in a symbiotic relationship with the bacteria (Simova et al., 2002; Witthuhn et al., 2004; Marsh et al., 2013). Three genera of yeasts are commonly isolated from kefir grains or milk, and typically make up the majority of the total yeast population; *Saccharomyces*, *Kluyveromyces*, and *Candida* (Angulo et al., 1993; Marquina et al., 2002; Simova et al., 2002; Diosma et al., 2014).

Many different species of *Saccharomyces* have been isolated from kefir, however, *S. cerevisiae* and *S. unisporus* are the most common and present in many varieties (Angulo et al., 1993; Marquina et al., 2002; Latorre-García et al., 2007; Diosma et al., 2014). *Kluyveromyces* make up the majority or entirety of the lactose utilizing yeast population, with *K. marxianus* and *K. lactis* being the two most common species (Simova et al., 2002; Latorre-García et al., 2007; Diosma et al., 2014). The *Candida* population is made up of a wide range of species with *C. holmii* and *C. kefyr* being the most prevalent (Angulo et al., 1993; Marquina et al., 2002). Outside of these three genera, only *Pichia* has been identified with any regularity and in each case the species was identified as *Pichia fermentans* (Angulo et al., 1993; Wang et al., 2008). As fermentation progresses the proportions of some yeast species change with non-lactose fermenting yeasts, such as *Saccharomyces*, decreasing, whereas lactose utilizing *K. marxianus* and *K. lactis* show a similar distribution between grain and kefir (Simova et al., 2002).

Unlike the bacterial population in kefir grain, the yeast component of the grain fluctuates considerably between grains when analyzed using culture independent techniques. Despite this, a small number of yeasts such as *Kazachstania*, *Kluyveromyces*, and *Naumovozyma* tend to be the dominant genera present in both the grain and fermented milk (Zhou et al., 2009; Marsh et al., 2013; Vardjan et al., 2013; Garofalo et al., 2015; Korsak et al., 2015). Of these main genera, only *Naumovozyma* has not been isolated in culture based studies. *Kazachstania unispora*, the species of *Kazachstania* present is also known as *Saccharomyces unisporus* (Marsh et al., 2013). Sequencing based approaches have also identified over a dozen yeast species that had not previously been associated with kefir, such as *Dekkera anomala*, *Issatchenkia orientalis*, and *Pichia fermentans*, and have even shown that, in some grains, the yeast population is dominated by a mix of these other species (Marsh et al., 2013; Garofalo et al., 2015). **Table 1** contains a complete list of yeast species found in culture dependent and culture independent studies.

### Culture Dependent vs. Culture Independent Methods

As expected, sequencing based methods often identify organisms that are not readily isolated by traditional culture based methods. This may be due to the presence of these organisms in extremely low numbers, or some of these organisms may be unable to grow on traditional media due to the complex symbiotic relationships present in kefir. Indeed, this may account for why certain *Lactobacillus* species have only been identified in sequencing based studies (Dobson et al., 2011). For example *L. kefiranofaciens* has not consistently been isolated in culture based methods but is regularly identified as a major part of the *Lactobacillus* population present in kefir when culture independent methods are used which may be due to the more strict anaerobic nature of this species when compared to other *Lactobacillus* species (Wang et al., 2012). While sequencing based methods have proven to be very valuable for identifying difficult to culture organisms, high throughput sequencing of 16S amplicons are limited with respect to their ability to consistently identify organisms at the species level (Marsh et al., 2013). Additionally, with metagenomic analyses there is the possibility that population dynamics may be skewed if there are dead cells present. While large numbers of dead cells from one species may indicate the importance of that species to kefir, the detection of these dead cells can still be problematic at later times during fermentation as they would not be actively involved in the community at these time points. Culture based methods remain essential as they allow organisms to be phenotypically tested. Regardless, the advent of sequence based technologies has increased the knowledge of which organisms are present in kefir grains and fermented milk and will allow for the development of new strategies to facilitate the isolation of organisms previously overlooked.

## Cholesterol Metabolism and ACE Inhibition

Due to the highly complex microbiota of kefir, there is a multitude of organisms and metabolic products present in the fermented milk. This combination of live microbial organisms and metabolites contributes to a wide range of effects attributed to kefir many of which are health benefits. Cardiovascular disease (CVD) is one of the leading causes of death in the western world, with high levels of serum cholesterol being a major risk factor for the disease. Diet can play a major role in the management of serum cholesterol levels and thus, ones risk of contracting CVD (WHO, 1982). It has been shown that milk and especially fermented milks are able to reduce serum cholesterol levels in animal trials (Beena and Prasad, 1997; Sibel Akalin et al., 1997). Kefir grains are capable of reducing the cholesterol levels of milk through the fermentation process and have been shown to reduce the levels of cholesterol present by between 41 and 84% after 24 h fermentation and a further 48 h of storage (Vujičić, 1992). While cholesterol reduction varied from one grain to another, these differences did not reflect the country of origin of the grain; Yugoslavian grains had both the highest and lowest levels of cholesterol reduction. Single kefir isolates have also been shown to assimilate cholesterol, with *K*. *marxianus* being one of the more effective. When *K. marxianus* strains K1 and M3 were inoculated in broth supplemented with cholesterol for 20 h, cholesterol levels decreased 70–99% (Liu et al., 2012). These same strains of *K. marxianus* showed significant levels of bile salt hydrolase (BSH) activity which were proportional to the rate of cholesterol lowering (Liu et al., 2012). BSH deconjugates bile acids and, as deconjugated bile salts is less soluble and less efficiently reabsorbed from the intestinal lumen, this leads to increased bile salt excretion in the faces (Zhuang et al., 2012). BSH deconjugation contributes to cholesterol lowering abilities of kefir as cholesterol is utilized in bile acid synthesis.

Cholesterol lowering properties of kefir have been validated in animal models. In a study using male golden Syrian hamsters fed cholesterol free or cholesterol enriched diet, both milk kefir and soyamilk kefir reduced serum triacylglycerol and total cholesterol while improving the atherogenic index (i.e., ratio of non-HDL-cholesterol to HDL-cholesterol). The cholesterol lowering effect was independent of whether the hamsters were fed the cholesterol free or cholesterol enriched diet (Liu et al., 2006a) indicating that kefir feeding altered endogenous cholesterol metabolism. Concentrations of cholesterol in the liver were also observed to decrease in both milk kefir and soyamilk kefir fed hamsters, and the secretion levels of fecal bile acid and cholesterol significantly increased for both groups. The increase in fecal bile acid is likely a result of the deconjugation of bile acid by microbes present in the kefir, while the higher levels of cholesterol secretion were likely due to the inhibition of cholesterol absorption in the small intestine due to the binding and assimilation of cholesterol by these same microbes (Xiao et al., 2003).

*Lactobacillus plantarum* MA2 isolated from kefir has also shown hypocholesterolemic activity in male Sprague-Dawley (SD) rats fed a high cholesterol diet. Rats fed a diet supplemented with this organism had significantly lower total serum cholesterol, LDL-cholesterol, triglycerides, liver cholesterol and triglycerides in conjunction with increased fecal cholesterol secretion (Wang et al., 2009). A similar study that used a high cholesterol diet supplemented with *L. plantarum* strains Lp09 and Lp45 in SD rats found that these strains had the same effect (Huang et al., 2013a). Huang et al. (2013b) also found that *L. plantarum* Lp27 was able to decrease serum total cholesterol, LDL-cholesterol, and triglycerides in hypercholesterolemic SD rats that consumed a diet supplemented with Lp27. A proposed mechanism for decreased serum cholesterol is the inhibition of cholesterol absorption. The Niemann-Pick C1-like 1 (NPC1L1) gene, which plays a critical role in the absoption of cholesterol (Altmann et al., 2004), is down-regulated in rats fed Lp27 and in *in vitro* tests with Caco-2 cells (Huang et al., 2013b). Zheng et al. (2013) found that *L. acidophilus* LA15, *L. plantarum* B23, and *L. kefiri* D17 were all able to lower serum total cholesterol, LDL, and triglyceride levels in SD rats fed a high cholesterol diet. The three strains also increased fecal cholesterol and bile acid secretion (Zheng et al., 2013). *K*. *marxianus* YIT 8292 was also shown to decrease plasma and liver cholesterol levels in addition to increasing fecal sterol and bile acid excretion and the concentration of short chain fatty acids in the cecum (Yoshida et al., 2005), indicating that both bacteria and yeast can contribute to this characteristic. This effect was shown to be specific to α-mannan and β-glucan present in the cell wall of *K. marxianus* (Yoshida et al., 2005). In addition to individual microbes in kefir having an ability to reduce cholesterol, kefiran has also been shown to improve cholesterol and blood pressure levels. In a study using spontaneously hypertensive and stroke prone (SHRSP/Hos) rats fed a high fat diet, kefiran supplementation reduced serum total cholesterol, serum LDL-cholesterol, serum triglycerides, liver cholesterol, and liver triglycerides (Maeda et al., 2004b), however, the concentrations used for kefiran supplementation were not discussed. Decreases in the blood pressure and angiotensin converting enzyme (ACE) activity were also observed. ACE inhibitory action has been attributed to commercial kefir made from caprine milk when tested *in vitro*, with the mode of action being attributed to two small peptides released from casein during the fermentation process (Quiros et al., 2005).

In contrast to these studies, St-Onge et al. (2002) found that when mildly hypercholesterolemic men consumed kefir as part of their diet for 4 weeks there was no significant change to total serum cholesterol, LDL-cholesterol, HDL-cholesterol, or triglyceride concentrations. They did note an increase in fecal bacterial counts and short chain fatty acid levels, including propionic acid. Additionally, a study examining Wistar rats fed a standard diet supplemented with kefir for 22 days found no significant differences in serum cholesterol when compared to rats on a control diet (Urdaneta et al., 2007). While these two studies seem to conflict with other findings, this may be in large part due to the fact that different kefir grains were used for each of these studies. Additionally, the aforementioned Liu et al. (2006a) study had a timeline of 8 weeks, while St-Onge et al. (2002) and Urdaneta et al. (2007) had timelines of 4 weeks and 22 days, respectively. It may be significant that, in the study of hypercholesterolemic men, an increase in fecal propionic acid was noted. Propionic acid has been shown to inhibit acetate incorporation in to triacylglycerol and plasma cholesterol (Wolever et al., 1995). Thus, a hypocholesterolemic effect may have been observed had the study continued for a longer time period.

## Effects on the Host Gut and Gut Microbiome

### Pathogen Exclusion

One of the main ways through which probiotic-containing food products can exert beneficial effects is altering the gut microbiota. This can be done either through the introduction of new species or strains in to the gastrointestinal tract, or by promoting the growth of beneficial microbes which are already present. Some examples are presented here. In multiple studies, consumption of kefir or kefiran in an animal model has been associated with an increase in microbes thought of as beneficial, such as *Lactobacillus* and *Bifidobacterium*, while simultaneously decreasing harmful microbial species such as *Clostridium perfringens* (Liu et al., 2006b; Hamet et al., 2016). Kefir consumption was also able to reduce the severity of *Giardia intestinalis* infection in C57BL/6 mice, with the reported mechanism being through modulation of the immune system (Correa Franco et al., 2013). Furthermore, specific strains of *Lactobacillus* isolated from kefir have been shown to adhere to Caco-2 cells and inhibit the adherence of *Salmonella typhimurium* and *Escherichia coli* O157:H7 (Santos et al., 2003; Hugo et al., 2008; Huang et al., 2013a). The ability of these *Lactobacillus* species to bind to Caco-2 cells illustrates a likely mechanism for the increase in *Lactobacillus* species observed in the fecal microbiota of rats fed kefir (Liu et al., 2006b; Carasi et al., 2015). In an *in vivo* study where BALB/c mice were intragastrically challenged with *E. coli* O157:H7, mice receiving *L*. *kefiranofaciens* M1 prior to *E. coli* challenge showed reduced symptoms of infection, including intestinal and renal damage, bacterial translocation, and Shiga toxin penetration as well as increased EHEC-specific mucosal IgA responses (Chen et al., 2013)

Other *in vitro* work has also shown that lactobacilli isolated from kefir have the ability to protect Vero cells from type II Shiga toxin produced by *E. coli* O157:H7, leading to lower levels of cell death (Kakisu et al., 2013). Similar effects were apparent in another study where they observed that kefir fermented milk inhibited the ability of *Bacillus cereus* extracellular factors to cause damage to Caco-2 cells (Kakisu et al., 2007).

As well as regulating microbial composition, kefir can alter the activity of the microbiota. Certain *Bifidobacterium* strains have been shown to exhibit increases in growth rate when cultured in kefir and changes in gene expression have also been observed (Serafini et al., 2014). These changes in gene expression resulted in increased expression levels of multiple genes associated with *pil3*, a sortase dependent pilus that has been shown to be extremely important for interaction with the host endothelial cells and is especially important for adherence and modulation of the host inflammatory response (Turroni et al., 2013; Serafini et al., 2014). While this specific example shows the potential positive effects kefir can have on existing organisms within the gut microbiota, it is still unclear as to how this translates to the complex population of the whole microbiome.

### Antibacterial and Antifungal Properties

Kefir, and kefir associated strains, has shown a multitude of antibacterial and antifungal activities (**Table 2**). Kefir fermented milk has been tested in disk diffusion experiments against a wide range of pathogenic bacterial and fungal species and found to have antimicrobial activity equal to ampicillin, azithromycin, ceftriaxone, amoxicillin, and ketoconazole against many of these species (Cevikbas et al., 1994; Yüksekdağ et al., 2004; Rodrigues et al., 2005; Huseini et al., 2012).

**Table 2:** List of pathogenic organisms that kefir or kefir-associated organisms have demonstrated antimicrobial effects against.

Microbial species	Reference
**Bacteria**	
*Staphylococcus aureus*	Cevikbas et al., 1994; Ryan et al., 1996; Yüksekdağ et al., 2004; Rodrigues et al., 2005; Miao et al., 2014; Leite et al., 2015; Zanirati et al., 2015
*Pseudomonas aeruginosa*	Cevikbas et al., 1994; Ryan et al., 1996; Yüksekdağ et al., 2004; Rodrigues et al., 2005; Huseini et al., 2012; Zanirati et al., 2015
*Salmonella typhimurium*	Santos et al., 2003; Rodrigues et al., 2005; Golowczyc et al., 2008; Zanirati et al., 2015
*Escherichia coli*	Ryan et al., 1996; Santos et al., 2003; Yüksekdağ et al., 2004; Rodrigues et al., 2005; Golowczyc et al., 2008; Leite et al., 2015; Zanirati et al., 2015
*Salmonella enteritidis*	Santos et al., 2003; Miao et al., 2014
*Listeria monocytogenes*	Ryan et al., 1996; Santos et al., 2003; Rodrigues et al., 2005; Likotrafiti et al., 2015; Leite et al., 2015; Zanirati et al., 2015
*Bacillus subtilis*	Cevikbas et al., 1994; Ryan et al., 1996
*Salmonella enterica*	Golowczyc et al., 2008; Miao et al., 2014; Leite et al., 2015
*Enterococcus faecalis*	Ryan et al., 1996; Zanirati et al., 2015
*Shigella flexneri*	Santos et al., 2003
*Clostridium difficile*	Rea et al., 2007
*Klebsiella pneumonia*, *Proteus vulgaris*	Cevikbas et al., 1994
*Streptococcus pyogenes*, *Staphylococcus salivarius*	Rodrigues et al., 2005
*Bacillus cereus*, *Clostridium sporogenes*, *C. tyrobutyricum*, *Enterococcus faecium*, *Listeria innocua*, *Salmonella typhi*	Ryan et al., 1996
*Salmonella gallinarum*, *Shigella sonnei*	Golowczyc et al., 2008
*Bacillus thuringiensis*, *Shigella dysenteriae*	Miao et al., 2014
**Fungus**	
*Candida albicans*	Rodrigues et al., 2005
*Yersinia entocolitica*	Santos et al., 2003
*Aspergillus flavus*, *A. niger*, *Rhizopus nigricans*, *Penicillium glaucum*	Miao et al., 2014
*Staphylococcus epidermidis*, *Candida stellatoidea*, *C. tropicalis*, *C. krusei*, *Saccharomyces cerevisiae*, *Rhodotorula glutinis*, *Torulopsis glabrata*	Cevikbas et al., 1994

In addition to the antimicrobial effects of kefir fermented milk as a whole, there are also specific microbes which exert antimicrobial properties on their own. For instance, *L. plantarum* ST8KF produces the bacteriocin ST8KF which exhibits antimicrobial action against *Enterococcus mundtii* and *Listeria innocua* (Powell et al., 2007). Other kefir-derived *Lactobacillus* species such as *L. acidophilus* and *L. kefiranofaciens*, as well as some *S. thermophilus* strains have shown antimicrobial activity against a whole range of pathogenic organisms including *E. coli, L. monocytogenes, S. aureus, S. typhimurium, S. enteritidis, S. flexneri*, *P. aeruginosa*, and *Y. enterocolitica* when tested using an agar spot test (Santos et al., 2003; Yüksekdağ et al., 2004; Golowczyc et al., 2008). Other kefir lactobacilli have also shown antimicrobial activity in *in vitro* tests against *S. typhimurium*, and *E. coli* that have already adhered to Caco-2 cells (Golowczyc et al., 2008). Lacticin 3147 is produced by a strain of *L. lactis* isolated from kefir and has an extremely broad range of antimicrobial activity, affecting *B. cereus*, *B. subtilis*, *C. sporogenes*, *C. tyrobutyricum*, *Enterococcus faecium*, *E. faecalis*, *L. innocua*, *L. monocytogenes*, *S. aureus*, and *C. difficile* (Ryan et al., 1996; Rea et al., 2007). Another bacteriocin of kefir origin is F1, which is produced by the *Lactobacillus paracasei* subsp. *tolerans* strain FX-6 source from a Tibetan kefir grain. F1 has been shown to inhibit a wide range of bacterial and fungal species including *S. aureus*, *Shigella dysenteriae*, and *Aspergillus niger* (Miao et al., 2014). *L*. *kefiri* B6 isolated from kefir was also capable of inhibiting and inactivating *L. monocytogenes* when in the presence of galactooligosaccharide *in vitro*, however, this effect was not observed with *E. coli* and, in this case, further investigation of the mechanism of this inactivation is needed (Likotrafiti et al., 2015). Similarly, Leite et al. (2015) isolated multiple strains of *L. lactis* and *Lb. paracasei* from kefir capable of producing bacteriocin-like substances that were inhibitory to *E. coli*, *S. enterica*, *S. aureus*, and *L. monocytogenes*, however, more work is needed in order to better characterize these substances and determine the range of their antimicrobial activity as well as their novelty. In a study examining LAB isolated from Brazilian kefir grains, *L. kefiranofaciens* 8U showed the ability to inhibit multiple pathogens including *P. aeruginosa*, *L. monocytogenes*, and *E. faecalis in vitro*, but again more work is needed in order to determine the mechanism behind this inhibition (Zanirati et al., 2015).

## Antitumor Effects

Kefir also has significant antitumor activity against multiple cancer cell types. *L*. *kefiri* was shown to increase apoptosis of multiple drug resistant human myeloid leukemia cells *in vitro* through the activation of caspase 3 in a dose dependent manner (Ghoneum and Gimzewski, 2014). The cell free fraction of kefir has shown antitumor activity *in vitro* when it was observed to have a dose dependent anti-proliferative effect on the gastric cancer cell line SGC7901 (Gao et al., 2013). This study further demonstrated that cell free kefir was able to induce apoptosis in SGC7901 cells through up regulation of the *bax* gene, and apoptosis promoter and anti-oncogene, and down regulation of the *bcl-2* gene, which is an apoptosis inhibitor and known oncogene (Sorenson, 2004). In addition to the promotion of cell death in cancerous cells, antimutagenic effects have been demonstrated in studies with known carcinogens such as methylmethanosulphate, methy-lazoxymethanol, sodium azide, aflatoxin B1, and 2-aminoanthracene as indicated by the Ames test (Guzel-Seydim et al., 2006).

In mouse models of fusiform cell sarcomas, mice receiving intraperitoneal kefir had reduced tumor size, with some tumors completely disappearing over a 20 days treatment period (Cevikbas et al., 1994). While this is impressive, it has yet to be determined if these findings can be replicated in the case of oral consumption. A separate study utilizing a murine breast cancer model showed that kefir feeding prior to challenge with the tumor resulted in decreased size and increased apoptosis of the tumor, and that the levels of IgA+ cells and CD4+ T cells were also increased (de Moreno de LeBlanc et al., 2007). Mice with breast cancer tumors fed kefir also showed increased serum levels of Il-10 and IL-4 (de Moreno de LeBlanc et al., 2006). These studies both showed increases in immune cell populations and recruitment, pointing to a possible mechanism for the reduction of tumor size. These findings are consistent with other studies that have shown that kefir is able to modulate the immune system in the gut and show that the immunomodulatory abilities of kefir may not be limited to the gastrointestinal tract (Thoreux and Schmucker, 2001; Vinderola et al., 2005; Correa Franco et al., 2013).

## Wound Healing

The antimicrobial properties of kefir may lead to its use for non-traditional applications. Indeed, when rats bearing open wounds inoculated with *S. aureus* were treated with a gel made from kefir grains, it was found that the wounds healed at a much faster rate than was observed in control rats that received no treatment or rats that received a traditional treatment of 5 mg/kg neomycin-clostebol emulsion (Rodrigues et al., 2005). Gels made from kefir and kefir grains were found to be more effective at reducing wound size in *P. aeruginosa* contaminated third degree burns than a traditional silver sulfadiazine treatment in a rat model of burn wounds (Huseini et al., 2012; Rahimzadeh et al., 2014). Furthermore, a study using a rabbit model for contaminated open wound also found that gel made from kefir grain resulted in quicker healing times and quicker clearing of infection (Atalan et al., 2003).

These decreased healing times are likely due to multiple factors. One such factor is the ability of kefir to inhibit the growth of bacterial and fungal cells, thus leading to a cleaner wound, as shown to be the case in some studies (Atalan et al., 2003; Huseini et al., 2012). Another possible factor is the ability to modulate the immune system and recruit immune cells to help with the healing process.

## Immunomodulatory Effects

One of the major ways probiotic products such as kefir are able to produce health benefits is through the modulation of the gastrointestinal immune system. When young rats inoculated intra-duodenally with cholera toxin (CT) were fed kefir, the levels of anti-CT IgA in the serum increased as did the secretion levels of anti-CT IgA in the Peyer’s Patches, the mesenteric lymph nodes, the spleen, and the intestinal lamina propria compared to CT alone (Thoreux and Schmucker, 2001). This same effect, however, was not observed in older mice that underwent the same treatment, suggesting that whatever mechanism is responsible for the observed change in the young rats is either no longer present in the senescent mice or requires a much larger dosage of kefir in order to activate it. Additional studies in to the mechanism as well as investigations with middle aged mice are needed to provide further insight in to this phenomenon. In an infection of C57BL/6 mice with *G*. *intestinalis*, kefir consumption reduced intensity of infection by mitigating the ability of *G. intestinalis* to suppress the mounting of an inflammatory response. This impact was mediated through increases in the levels of TNF-α and IFN-γ expression and through higher levels of IgA positive and RcFc𝜀 positive cells (Correa Franco et al., 2013). There have also been studies showing increases in IgA and IgG+ cells in the small intestine of rats that were fed both regular and pasteurized kefir, as well as increases in the levels of IL-4, IL-10, IL-6, and IL-2 positive cells in the lamina propria of these same rats. Increases were also seen in anti-inflammatory cytokines such as IL-10, IL-4, and IL-6, all of which promote a Th2 response (Vinderola et al., 2005). Interestingly, increases in IFN-γ, TNFα, and IL-12 (all of which are pro-inflammatory and promote a Th1 response) were observed only in rats fed pasteurized kefir. The increase in pro-inflammatory cytokines in the pasteurized kefir groups was likely due to the reduced cell wall integrity of heat killed cells exposing more inflammatory microbial products. The fact that pasteurized kefir was able to elicit an effect shows that the mechanisms behind this immune modulation are not entirely dependent on live cells, and may be due to metabolites present in the kefir (Iraporda et al., 2014). However, it should be noted that in this study live cells had a generally more substantial impact as live kefir was able to confer a similar effect at 1/10 the concentration and without eliciting a pro-inflammatory immune response (Vinderola et al., 2005).

When fed to mice over 2–7 days, solid fractions of kefir that contained live bacteria have been shown to increase the levels of IFN-γ, TNF-α, and IL-6 in peritoneal macrophages as well as to increase the levels of IL-1α, IL-10, and IL-6 in adherent cells isolated from the Peyer’s patch of mice (Vinderola et al., 2006b). IFN-γ and TNF-α increased early in feeding, however, they quickly decreased back to control levels by day 7 along with IL-1α while IL-6 and IL-10 levels remained high through the 7 days feeding period (Vinderola et al., 2006b). *In vitro* experiments with lactobacilli isolated from kefir have shown that they induce higher secretion levels of IL-1β, IL-6, TNF-α, IL-10, IL-8, and IL-12 in peripheral blood mononuclear cells and are able to decrease the ccl20 response in Caco-2 cells to TLR agonists such as bacterial flagella, with largely different effects being observed for different strains of lactobacilli tested (Carasi et al., 2015). In general, strains of *L. kefiri* that induced lower TNF-α/IL-10 and higher IL-10/IL-12 ratios showed a much greater decrease in the pro-inflammatory response of ccl20 to stimulation with bacterial flagella, indicating the importance of IL-10 in promoting a Th2 response while simultaneously inhibiting the pro-inflammatory Th1 response. Mice that were fed *L. kefiri* for a period of 21 days showed altered gene expression profiles in the ileum, colon, Peyer’s Patches, and mesenteric lymph nodes, with proinflammatory cytokines such as IFN-γ and IL-23 being down regulated and IL-10 being up regulated (Carasi et al., 2015). This further indicates that lactobacilli isolated from kefir have the ability to supress the production of pro-inflammatory cytokines while promoting anti-inflammatory cytokine production. *L*. *kefiranofaciens* co-incubation with mouse macrophage cells decreased the levels of pro-inflammatory cytokines IL-1β, and IL-12 while simultaneously increasing the level of the anti-inflammatory cytokine IL-10, which acts to specifically inhibit the production of IL-12 and IL-1β (Hong et al., 2009). Additionally, *L. kefiranofaciens* was able to ameliorate colitis in a DSS induced mouse model and enhance Th1 responses to TLR agonists in germ free mice by increasing the production of IFN-γ and IL-12 upon stimulation (Chen and Chen, 2013). Further investigation into the mechanisms of protection against colitis showed that *L. kefiranofaciens* M1 decreased the production of pro-inflammatory cytokines IL-1β and TNF-α, while increasing the production of IL-10 *in vivo* (Chen et al., 2012). This effect was also TLR-2 dependent as *L. kefiranofaciens* M1 was unable to improve DSS colitis in TLR-2 knockout mice (Chen et al., 2012).

The cell free fraction of kefir is also capable of modulating the immune system, and has been shown to modulate innate immune responses *in vitro* by lowering the activation of Caco-2-ccl20:luc cells that had been stimulated by *Salmonella* flagellar protein FliC, IL-1β, or TNF-α (Iraporda et al., 2014). One of the likely mechanisms was revealed when it was found that a 100 mM lactic acid solution at pH 7 was able to elicit a comparable level of immune modulation in FliC stimulated cells when preincubated with the solution (Iraporda et al., 2014). The lactic acid solution was also found to lower the level of NFκ-B activation in Caco-2 cells stimulated with FliC and was even able to down regulate the expression of pro-inflammatory cytokines ccl20, IL-8, CXCL 2, and CXCL 10 without affecting genes involved in the normal function of enterocytes (Iraporda et al., 2014). These results indicate just how important the metabolites produced during fermentation are to the ability of kefir to elicit beneficial responses or effects in the host.

In general studies using whole kefir, kefir fractions, or organisms isolated from kefir found that whether tested *in vitro* or *in vivo*, the result was a shift from a Th1 immune response to a Th2 response as well as increases in the levels of IgA present (Thoreux and Schmucker, 2001; Vinderola et al., 2005, 2006b; Hong et al., 2009; Carasi et al., 2015). The only study which seems to show a consistently increased Th1 response was conducted with germ free mice, while all other studies used conventional mice or rats (Chen and Chen, 2013). This may account for the difference in findings as it is quite possible that the observations from the germ free mice had more to do with the introduction of a bacterial population to the gut than it did with the specific bacterial species that comprised that population. The fact that most studies also observed increases in some pro-inflammatory cytokines such as TNF-α, IFN-γ, or IL-12 may be explained by an initial reaction of the immune system to common TLR agonists present, which was ultimately supressed following further interaction with the immune cells of the GI tract.

## Anti-Allergenic Effects

Allergic diseases have been on the rise in the developed world for decades, leading to higher incidences of conditions such as asthma and food allergy (Yazdanbakhsh et al., 2002). Many allergies, especially those related to food, are developed early in life, with the majority of food allergies developing within the first 2 years of life (Wood, 2003). Although most food allergies developed early in life do not persist, some can become lifelong conditions (Wood, 2003). Recent work has shown that an increasingly important factor in determining if a child develops allergic disease, be it food allergy or asthma, is the level of complexity and the specific organisms present in the gut microbiota (Kirjavainen et al., 2002; Sjogren et al., 2009; Azad et al., 2013; West, 2014). Higher levels of *Bifidobacterium* and group 1 lactobacilli (obligate heterofermentative lactobacilli such as *L. acidophilus, L. delbrueckii, and L. helveticus*) in the gut of infants have been associated with a lower incidence of allergic disease later in life (Sjogren et al., 2009), and both kefir and kefiran have been observed to exert these effects on the gut microbiota in animal trials (Liu et al., 2006b; Hamet et al., 2016). Supplementation with *Bifidobacterium* has been shown to influence the intestinal microbiota of weaning infants by reducing levels of *Bacteroides* and has been associated with lower incidence of food allergy (Kirjavainen et al., 2002). Studies with antibiotics in the early life period have also highlighted the importance of appropriate microbial stimulation of the immune system for protection against asthma development (Russell et al., 2012).

One of the main mechanisms behind food allergy is an imbalance in the Th1/Th2 cell ratio, leading to a heightened IgE response (Tanabe, 2008). Studies of *in vitro* reactions of human monocytes with a probiotic made up of multiple LAB showed that exposure to these LAB resulted in a much higher IFN-γ/IL-4 ratio, similar to what would be seen during a Th1 response (Tsai et al., 2012). In addition to the *in vitro* studies carried out, Tsai et al. (2012) found that both total IgE and OVA-specific IgE were significantly lower in mice that had been sensitized to OVA (ovalbumin) and then fed a LAB mixture than in control mice which had also been sensitized to OVA but did not receive any LAB mixture. Studies such as this indicate that kefir may help relieve some allergy symptoms.

In a study utilizing an ovalbumin sensitization mouse asthma model, it was found that mice receiving intra-gastric kefir showed lower levels of airway hyper-responsiveness (AHR) than control mice, and, impressively, had lower levels of AHR than the positive control group receiving an anti-asthma drug (Lee et al., 2007). This same study found that mice receiving kefir exhibited significantly lower levels of eosinophil infiltration in the lung tissue as well as in the brochoalveolar lavage fluid (BALF). These mice also showed lower levels of IgE, IL-4, and IL-13 in the BALF, all of which are associated with the Th2 response which is responsible for allergic reaction (Lee et al., 2007). It has also been found that oral feeding of kefir in OVA sensitized mice resulted in significantly lower levels of anti-OVA serum IgE and IgG1 antibodies than those found in mice given water or unfermented milk (Liu et al., 2006b). Studies examining the *in vitro* effect of heat-killed lactobacilli isolated from kefir on mouse peritoneal macrophages showed that even after being heat-inactivated, the lactobacilli were able to induce the expression of Th1 cytokines such as IFN-γ, TNF-α, IL-12, and IL-1β (Hong et al., 2010). These same heat-inactivated lactobacilli also reduced the levels of anti-OVA IgE in the serum when fed orally to OVA sensitized mice, while increasing the expression of IL-12 and decreasing the expression of IL-5 in splenocytes. An increase in the levels of regulatory T-cells was also detected in these mice (Hong et al., 2010). In a study of OVA sensitized mice fed with heat-inactivated strain M1 of *L. kefiranofaciens*, the inactivated M1 was able to decrease levels of pro-inflammatory and Th2 cytokines such as IL-4, IL-6, IL13, and ccl20 in both the splenocytes and BALF of the mice while decreasing OVA-specific IgE and the Th17 associated cytokine IL-17, both of which are strongly associated with an asthmatic response. The M1 treatment was also able to increase the levels of regulatory T cells present (Hong et al., 2011).

While all of these studies reveal a consistent pattern, it is interesting to note that many of the cytokine profiles are in stark contrast to those found in studies without antigen sensitization or challenge. This highlights both the complexity of the immune system and the need for a balance between the different possible reactions such as the Th1 and Th2 responses. The fact that kefir can induce shifts in the immune system in both directions is promising as it may mean that the organisms in kefir are capable of regulating this balance in the immune system. This may be in part due to the increased number of regulatory T-cells observed in some of these studies, as regulatory T-cells play an important role in maintaining tolerance and supressing unnecessary inflammatory immune responses (Sakaguchi, 2011).

## Health Benefits of Yeast in Kefir

As noted above, one unique characteristic of traditionally produced kefir relative to many other commercially produced fermented dairy products is the presence of a large population of yeast in both the kefir grain and in the fermented milk (Marsh et al., 2013). Although the majority of commercialized probiotic microbes are bacteria such as lactobacilli and bifidobacteria, there are some yeast species and strains that have been recognized to have probiotic properties, such as *Saccharomyces boulardii* (Corthier et al., 1986; Czerucka et al., 2007). *S. boulardii* has been shown to improve the symptoms of *Clostridium difficile* associated diarrhea as well as reduce inflammation and alter the immune state and reactions in the gut, leading to its adoption as a treatment for *C. difficile* diarrhea (Buts et al., 1994; Castagliuolo et al., 1999; Kotowska et al., 2005; Villarruel et al., 2007).

Some yeasts from kefir have also shown immunomodulatory activities. For example *K*. *marxianus* B0399 has been shown to have the ability to adhere to Caco-2 cells (Maccaferri et al., 2012). When co-incubated with lipopolysaccharide (LPS) stimulated Caco-2 cells, a significant decrease in the secretion of IL-10, IL-12, IL-8, and IFN- γ was observed (Maccaferri et al., 2012). Additionally, *K. marxianus* B0399 elicited a decrease in the secretion of pro-inflammatory cytokines TNF-α, IL-6, and MIP-1α when co-incubated with PBMCs that had been stimulated with LPS (Maccaferri et al., 2012). This same study showed that in an *in vitro* colonic model system, *K. marxianus* was able to stably form a population in the model while simultaneously enhancing the levels of *Bifidobacterium*. Increases in the levels of the short chain fatty acids acetate and propionate were also observed. Utilizing a Caco-2 cell line with a ccl20 reporter gene, Romanin et al. (2010) were able to show that multiple yeast strains of *S*. *cerevisiae* (CIDCA 81109, 81106, 8112, 9127, 9123, 9136, 9133, 9124, 81103, 9132, 81108, 81102, 8175, and 8111), *K*. *marxianus* (CIDCA 81111, 8116, 8118, 81105, 8153, 8154, 8113, 81104, and 9121), and *Issatchenkia* spp. (CIDCA 9131) were able to inhibit the expression of the ccl20 reporter when incubated with the cells prior to stimulation with *Salmonella* flagellar protein FliC. From these yeasts, *K. marxianus* CIDCA 8154 was selected for further testing and showed the ability to inhibit the levels of ccl20 expression in Caco-2 cells regardless of whether the stimulation came from FliC, IL-1β, or TNF-α. The strain also inhibited the expression of IL-8 and MIP-2α in HT-29 cells and inhibited ccl20 expression in a mouse ligated intestinal loop model when administered prior to stimulation with FliC (Romanin et al., 2010). Yeasts isolated from kefir have also shown the ability to improve the probiotic properties of bacterial species by improving the viability of these bacterial strains over time in simulated gastric and intestinal juice, and through improving the adhesion of LAB to Caco-2 cells in an *in vitro* model. This effect is likely due to the co-aggregation of the two microbial species (Xie et al., 2012).

## Kefiran and the Cell Free Fraction of Kefir

In addition to the microbial populations present in kefir and other fermented probiotics, there are also fermentation products and other by-products of the metabolism of these microbes that possess bioactivity. Some of these by-products may have a profound effect on the host without the presence of the microbial population. Such a by-product is kefiran, the exopolysaccharide produced by *L. kefiranofaciens* during fermentation (Maeda et al., 2004b; Vinderola et al., 2006a). Mice fed kefiran dissolved in drinking water showed increases in the levels of IgA+ B cells, as well as increases in IL-6, IL-10, and IL-12 in the lamina propria of the small intestine after 7 days of feeding (Vinderola et al., 2006a). In a murine model of asthma using OVA sensitization, kefiran introduced intra-gastrically 1 h prior to challenge reduced levels of the Th2 cytokines IL-4 and IL-5 and lowered AHR when compared to OVA challenged mice that did not receive kefiran (Kwon et al., 2008). After the same period the study showed increases in serum levels of IL-4, IL-6, IL-10, and IFN-γ (Kwon et al., 2008). Addition of kefiran to a co-incubation of *B. cereus* culture supernatant and Caco-2 cell monolayer resulted in reduced cell detachment and greater mitochondrial activity, as well as negated the haemolytic effect of the *B. cereus* culture supernatant on human red blood cells (Medrano et al., 2008). Genetically diabetic (KKAy) mice fed kefiran were found to have decreasing levels of blood glucose throughout a 30 days examination while a control group was found to have constantly increasing and generally higher levels of blood glucose throughout the same timeline (Maeda et al., 2004a). Using SD rats as a model for constipation, it was also found that kefiran significantly improved the symptoms of constipation over the control group (Maeda et al., 2004a).

A water-soluble polysaccharide isolated from kefir grain (KGF-C) was shown to improve humoral immune response in mice against Sheep Red Blood Cells (SRBC). The levels of anti-SRBC cells isolated from the spleen of mice immunized with SRBC while being intubated with KGF-C was significantly higher than in control mice 4 days post immunization (Murofushi et al., 1986). However, this effect was not seen in nu/nu mice (no thymus or T cell population) immunized with SRBC, or in conventional mice immunized with thymus-independent antigens, indicating that the mechanism of action is likely through the T cell population (Murofushi et al., 1986). Sphingomyelin isolated from kefir has been shown to increase IFN-β secretion in human MG-63 cells when compared to commercial sphingomyelin and sphingosine (Osada et al., 1993).

Kefir cell-free supernatant (KCFS) has been shown to increase the levels of IFN-β, IL-6, IL-12, and TNF-α secreted by RAW 264.7 cells through a TLR2 dependent mechanism (Hong et al., 2009). Cell-free fractions of kefir have also been shown to increase the levels of these cytokines in peritoneal macrophages and adherent cells from the Peyer’s patches of mice (Vinderola et al., 2006b). In addition, KCFSs were found to have a significant impact on tumor size, apoptosis, and immune recruitment in a murine breast cancer model, resulting in increased apoptosis of tumor cells and increases in the CD4+ T cell population (de Moreno de LeBlanc et al., 2007). In *in vitro* studies utilizing human T-lymphotropic virus 1 (HTLV-1) positive HuT-102 Malignant T lymphocytes as a model for T cell leukemia, the KCFS was found to inhibit proliferation by up to 98% while simultaneously decreasing the transcriptional levels of TGF-α. These effects have also been observed in HTLV-1 negative malignant T cells with the same decrease in TGF-α transcription being observed (Rizk et al., 2009; Maalouf et al., 2011). In addition to anti-proliferative effects, KCFS was found to induce apoptosis in both HTLV-1 positive and negative malignant T cells through the up regulation of *bax* and down regulation of *bcl-2* in a dose dependent manner (Rizk et al., 2013).

## Conclusion

The purpose of this review has been to collate and summarize that which is known about the microbial composition of kefir and how this composition plays a role in the health benefits associated with kefir consumption. Kefir is a dynamic fermented dairy product with many different factors affecting the benefits associated with its consumption. These factors include the variable yeast and bacterial species present, as well as metabolites such as kefiran and other exopolysaccharides. While kefir has been associated with health benefits for 100s of years, the exact form of these benefits has, until recently, not been studied. The use of animal models and other *in vitro* analyses has allowed for the elucidation of how kefir positively impacts host health. Whole kefir, as well as specific fractions and individual organisms isolated from kefir, provide a multitude of positive effects when consumed. These range from improved cholesterol metabolism and wound healing, to the modulation of the immune system and microbiome, and even the potential alleviation of allergies and cancers. Further studies into the mechanisms behind these effects will allow scientists to better understand exactly how kefir and other fermented dairy products confer these benefits as well as how to harness these traits outside of kefir itself.

The wide range of potential health promoting effects of kefir could lead to a further expansion on the popularity of both traditional fermented kefir and products that are manufactured with kefir fractions or organisms. In order to fully exploit the beneficial characteristics of kefir, a more in-depth understanding of the composition of kefir is critical. With advances in metagenomic analysis through the development of high-throughput sequencing technology, this is a very realistic prospect. Armed with this knowledge, it should be possible to more readily isolate and examine the phenotypic characteristics of individual organisms present in a kefir blend while also providing a greater insight into the evolution of these organisms and how they became specialized to the kefir ecosystem. The additional knowledge gained can also provide crucial information relating to the mechanisms and exact agents responsible for beneficial effects that have been attributed to kefir (Atalan et al., 2003; Rodrigues et al., 2005; Huseini et al., 2012; Rahimzadeh et al., 2014).

The need for further research does not only apply to the mechanisms by which kefir consumption exerts these effects but also which organisms or parts of kefir are responsible for each benefit. By determining which organisms and metabolites are essential for each process, the possibility arises for the commercial manufacturing of kefir that is specifically designed to create the most profound effect in those that consume it. As it stands currently, the highly variable nature of the organisms and metabolites present in traditional kefir requires health claims to be verified individually in each grain and kefir beverage. The ability to combine the best possible strains of the best organisms from multiple sources of kefir would create the potential for greater benefits than have been previously observed, with a measure of control over these effects that has not been possible in traditional kefir.

## Author Contributions

BB wrote the review and compiled, figures, tables, and references. PC supervised, edited, and approved the review. BW supervised, edited, and approved the review.

## Conflict of Interest Statement

The authors declare that the research was conducted in the absence of any commercial or financial relationships that could be construed as a potential conflict of interest.
